# Traffic control and social distancing evidence from COVID-19 in China

**DOI:** 10.1371/journal.pone.0252300

**Published:** 2021-06-02

**Authors:** Shanlang Lin, Ruofei Lin, Na Yan, Junpei Huang

**Affiliations:** School of Economics and Management, Tongji University, Shanghai, China; Nanyang Technological University, SINGAPORE

## Abstract

We collected COVID-19 epidemiological and epidemic control measures-related data in mainland China during the period January 1 to February 19, 2020, and empirically tested the practical effects of the epidemic control measures implemented in China by applying the econometrics approach. The results show that nationally, both traffic control and social distancing have played an important role in controlling the outbreak of the epidemic, however, neither of the two measures have had a significant effect in low-risk areas. Moreover, the effect of traffic control is more successful than that of social distancing. Both measures complement each other, and their combined effect achieves even better results. These findings confirm the effectiveness of the measures currently in place in China, however, we would like to emphasize that control measures should be more tailored, which implemented according to each specific city’s situation, in order to achieve a better epidemic prevention and control.

## 1. Introduction

During the COVID-19 outbreak, China adopted a series of the most comprehensive, thorough, and rigorous control measures, such as the “lockdown” of its cities, and it achieved successful results. The COVID-19 outbreak occurred during the Spring Festival, and a large population flow increased the transmission speed of the virus, and added to the difficulty in preventing and controlling the epidemic. In order to control the further spread of the virus, on January 23, the city of Wuhan went into lockdown. On the same day, several provinces in the country launched a *first-level response to major public health emergencies*. Next, cities in Hubei province were locked down successively, and other provinces in China also started a first-level response one after another. They also gradually adopted traffic control measures such as road blockades, and suspending public transport, and introduced social distancing measures such as quarantining suspected cases, isolating confirmed cases, closing residential areas and so on. The key to controlling the spread of the virus was to effectively separate the infected and suspected infected from the non-infected population. It was necessary to implement these strict measures aimed at reducing human-to-human contact particularly in the light of the absence of universal access to effective vaccines and antiviral drugs. Evaluation of and reflection on the effects of these measures can contribute to the better sharing of China’s experience and can help the whole world to jointly fight the crisis. Theoretically, these prevention and control measures can reduce the transmission rate of the virus and mitigate the epidemic. This leads to the question: what is the role of the prevention and control measures taken in China during the COVID-19 epidemic, and is there heterogeneity in the effectiveness of these measures?

In this paper, we report our analysis of the city-daily COVID-19-related panel data from mainland China collected from January 1 to February 19, 2020 including confirmed cases, specific content and start date/time of the control measures, climate data, and Baidu migration data. Since China has taken unprecedented and strict cross-administrative traffic control measures in this epidemic, we divide the epidemic control measures implemented during the COVID-19 outbreak in China into two types: traffic control measures and social distancing measures, and empirically evaluate their effectiveness and regional heterogeneity.

First, we analyzed the effects of the two types of measures implemented in China, and we found that nationally, both traffic control and social distancing measures played an important role in controlling the epidemic. Despite the inevitable cost, the implementation of traffic control measures is still desirable. Second, we compared both measures, and found that traffic control is more effective than social distancing. Moreover, the two measures are complementary, and the combined effect of the two achieves better results; Finally, based on the severity of the epidemic, we divided all cities in China into high-risk, medium-risk and low-risk areas in order to conduct sub-sample estimations. The results show that although both measures play a significant role in the control of the epidemic nationally, they have no statistically significant effect in the low-risk areas. Therefore, it is unnecessary to implement such severe prevention and control measures on a large nationwide scale, without targeting specific areas. We contend that epidemic prevention and control interventions should be tailored to specific local and regional conditions.

This paper contributes to the extensive literature that exists on assessing the effectiveness and application of public health policies. Although there are several studies evaluating isolation [1, 2], quarantine [3, 4], travel restrictions [5–7], school closures [8, 9], vaccination [10], and other epidemic control measures, most studies applied mathematical modeling, numerical simulation methods or statistical correlation analysis, while econometric approaches for causality analysis are rarely applied to the evaluation of health policies. Since the outbreak of COVID-19, there have been several studies on this virus using econometric methods [11–13], which lays a good foundation for our research. This paper applies econometrics methods to test the practical effect of the prevent and control measures taken in China during the COVID-19 epidemic by constructing econometric models, utilizing observational data, parameter estimation and statistical inference.

The paper also contributes more generally to the growing literature on COVID-19. The existing literature focuses on the transmission characteristics, the use of transmission models to predict the epidemic scale, and how to control the spread of the virus [14–19]. China has taken extremely strict epidemic prevention measures in the COVID-19 outbreak, namely across administrative traffic control. Taking a different approach than the existing literature, we divided the series of measures taken in China into two categories, traffic control and social distancing, and empirically tested the effects of these measures to explore which is more effective in what areas.

## 2. Methodology

Apply econometric methods, we empirically tested the effects of the two prevention and control measures using Chinese panel data from January 1, 2020 to February 19, 2020. Econometric methods are often used to measure the impact of a variable on economic growth. Similar to the early development of COVID-19, economic output is generally showing exponential growth, and this growth rate may vary with policies or other conditions [12]. Therefore, it is appropriate to use the econometric approach to analyze the impact of prevention and control measures on the development of the epidemic. Compared with statistical methods such as the Pearson correlation coefficient to identify correlativity, the econometric approach focuses more on identifying the causal relationship between variables [20], that is, whether the prevention and control measures lead to the containment of the epidemic. According to the control measures classification of COVID-19 in China, the baseline estimation equation is as follows:

$$y_{it}=\alpha +\beta_{1}{measure}_{it}+\sum_{2}^{n}\beta_{k}X_{kit}+\delta_{c}+\delta_{t}+\varepsilon_{it}$$ (1)

Where, *y_it_* represents the actual cumulative case growth rate of city i in date t:

$${actual\;cumulative\;case\;growth\;rate}_{it}=({actual\;case}_{it}-{actual\;case}_{it-1})/{actual\;case}_{it-1}$$ (2)

When there are no cases on the current day: *actual case*_*it*−1_ = 0, let *actual cumulative case growth rate_it_* = 0.

Considering that the average incubation period of COVID-19 is 5.2 days [21], we take the fifth lead of reported cases as the proxy variable of the actual cases, namely:

$${reported\;case}_{it}={actual\;case}_{it+5}$$ (3)

Taking Chengdu, China as an example, the number of reported cumulative confirmed cases in this city on February 1, 2020 was 73. Considering the incubation period of 5 days, 73 should be the number of real cumulative cases five days previously, i.e., on January 27, 2020, while the number of real cases on February 1 should be the cumulative number of reported cases on February 6.

*measure_it_* represents the main explanatory variables, namely traffic control and social distancing and the total score of the two measures. α is intercept. *β*_1_ is the coefficient of the main explanatory variable, if *β*_1_<0 and is statistically significant, then it indicates that the strengthening of prevention and control measures can reduce the cumulative case growth rate, that is, the measures can play a positive role in the prevention and control of the epidemic. We constructed scoring data as a proxy variable for the prevention and control measures.

We collected data on 14 epidemic control measures (Table 1), which are subsequently classified into two types: traffic control and social distancing, each with a score of 1, and scoring continues until the measure is canceled. For example, on January 21, Shanghai began to implement "quarantining contacts for 14 days." Since this measure comes under “social distancing”, then the score for distancing in Shanghai from January 21 was 1. On January 24, Shanghai began to implement "closing part of indoor urban public places", since this measure also comes under “social distancing”, in this case, “social distancing” in Shanghai will have another 1 point added from January 24, and so on, and finally, the points for traffic control and social distancing will be added up separately.

**Table 1:** Scores for traffic control and social distancing.

Traffic Control	Social Distancing
	Closing all the public places
Suspending all the cross-city passenger transport	Closing part of the public places
Suspending part of the cross-city passenger transport	Closed management of all the community
Monitoring all the cross-city passenger transport	Closed management of part of community
Monitoring part of the cross-city passenger transport	Quarantining returnees from key epidemic area (Hubei) for 14days
Suspending all the public transport	Quarantining all the returnees for 14days
Suspending part of the public transport	Quarantining contact for 14days
	Isolating and testing the suspected

*X_k_* is control variables, including population density (popdensity), number of beds in medical institutions (bed), effective distance from Wuhan (distance), average temperature (AT), and relative humidity (RH) to control the city characteristics on the spread of the epidemic. *β*_2k_ is coefficient of the control variable. *δ_c_* and *δ_t_* represent region-fixed effect and time-fixed effect respectively. *ε_it_* is error term, we use cluster-robust standard error to estimate the standard deviation [22].

As for the data used in this paper, the specific content and implementation time of the prevention and control measures comes from the information or announcements issued by the prevention and control headquarters of the prefecture-level administrative districts; the cumulative confirmed cases comes from the official release of the *National Health and Health Commission*; population density, and number of beds in medical institutions comes from the *China City Statistical Yearbook*; average temperature and relative humidity comes from the *China weather website*. We referred to the approach of [23] to calculate the effective distance from Wuhan to each city. The inter-city passenger flow data used in the calculation are from the *Baidu Migration* website. The explanation of each variable is shown in Table 2.

**Table 2:** Variable explanation.

Attribute	Variable	Explanation	Date Source
explained variable	Rate	actual cumulative case growth rate	National Health and Health Commission
explanatory variable	Total Score	Total score of prevention and control measures	Prevention and Control Headquarters of each city
	Traffic Control	score of traffic control measures	
	social distancing	score of social distancing measures	
control variable	Popdensity	Population densuty	China City Statistical Yearbook
	Bed	Numbers of beds in medical institutions	
	Distance	Effective distance	Baidu Migration website http://qianxi.baidu.com/
	AT	Average Temperature	China weather website http://www.weather.com.cn/
	RH	Relative Humidity	

The data in this paper consists of balanced panel data from 279 prefecture-level cities in China, collected from January 1 to February 10, 2020. Descriptive statistics of related variables are shown in Table 3.

**Table 3:** Statistical description.

variable	sample size	mean	standard deviation	minimum value	maximum value
Rate	13,950	0.085	0.442	0	19
Total Score	13,950	3.729	3.912	0	10
Traffic Control	13,950	1.758	1.802	0	4
social distancing	13,950	1.972	2.252	0	6
Popdensity	13,950	0.082	0.070	.001	0.565
Bed	13,950	12,906	17,135	920	142,708
Distance	13,950	4.100	0.678	1.883	5.707
AT	13,950	3.789	9.066	-31.2	26.4
RH	13,950	71.203	17.142	6	102

## 3. Results

### 3.1 Are the measures effective?

Table 4 reports our baseline regression analysis results. The explanatory variable is the actual cumulative case growth rate. The explanatory variables in column (1) and column (2) are the total score of prevention and control measures, column (3) and column (4) contain the score of traffic control measures, and column (5) and column (6) show social distancing score. Columns (2), (4), and (6) introduce control variables based on columns (1), (3), and (5). The data in the first row of each variable is the regression coefficient, with standard errors reported in parentheses. “*” denotes a rejection of the null hypothesis *H*_0_:*β*_1_ = 0 at a significance level of 10%, “**” denotes a rejection of the null hypothesis at a significance level of 5%, and “***” denotes a rejection of the null hypothesis at a significance level of 1%. The results show that the coefficients of the total score, traffic control, and social distancing are significantly negative, regardless of whether control variables are added, which indicates that the total effect of the two measures, traffic control and social distancing all significantly reduce the cumulative case growth rate, effectively containing the spread of the epidemic.

**Table 4:** Baseline regression.

	(1)	(2)	(3)	(4)	(5)	(6)
Total Score	-0.0236***	-0.0220***				
	(0.0030)	(0.0030)				
Traffic Control			-0.0254***	-0.0234***		
			(0.0065)	(0.0065)		
Social Distancing					-0.0340***	-0.0319***
					(0.0040)	(0.0041)
Popdensity		0.0318***		0.0346***		0.0307***
		(0.0112)		(0.0112)		(0.0112)
Bed		-0.0134**		-0.0161**		-0.0118*
		(0.0065)		(0.0065)		(0.0065)
Distance		-0.0133***		-0.0129***		-0.0133***
		(0.0038)		(0.0038)		(0.0038)
average temperature		-0.0035***		-0.0039***		-0.0034***
		(0.0011)		(0.0011)		(0.0011)
relative humidity		-0.0015***		-0.0017***		-0.0015***
		(0.0003)		(0.0003)		(0.0003)
Observations	12,555	12,555	12,555	12,555	12,555	12,555
R-squared	0.035	0.041	0.032	0.038	0.036	0.042
Control Variables	NO	YES	NO	YES	NO	YES
Province FE	YES	YES	YES	YES	YES	YES
Time FE	YES	YES	YES	YES	YES	YES

Standard errors in parentheses

*** p<0.01

** p<0.05

* p<0.1.

### 3.2 Which measure is more effective?

We have proved the effectiveness of the two measures, but which measure works better? To ensure that the coefficients of the two variables are comparable, we transferred the three explanatory variables into dummy variables (Traffic Control_dummy and Social Distancing _dummy) according to the mean value of the explanatory variables. For example, when the traffic control score is greater than the mean value, the value of Traffic Control_Dummy is 1, and vice versa, and the econometric model is as follows:

$$y_{it}=\alpha +\beta_{11}{\_Traffic\;Control\_dummy}_{it}+\beta_{12}{\_Social\;Distancing\_dummy}_{it}+\sum_{2}^{n}\beta_{k}X_{kit}+\delta_{c}+\delta_{t}+\varepsilon_{it}$$ (4)

Table 5 reports the results. Columns (1) and (2) report the result of the comparison between the two measures. It can be seen that the effect of traffic control is better than social distancing. Further, we introduced the interaction terms of the two measures (Interaction terms) in columns (3) and (4) to explore the substitutive and complementary relationship between the two measures. This shows that the interaction terms are negative, which is the same sign as the main explanatory variable. That is, the two measures have a complementary relationship and the joint effort of the two measures is more effective in the prevention of the spread.

**Table 5:** Comparison of the two measures.

	(1)	(2)	(3)	(4)
Traffic Control_dummy	-0.1066***	-0.1079***	-0.0818***	-0.0876***
	(0.0186)	(0.0202)	(0.0215)	(0.0233)
Social Distancing _dummy	-0.0519***	-0.0639***	0.0117	-0.0131
	(0.0179)	(0.0193)	(0.0330)	(0.0352)
Interaction terms			-0.0868**	-0.0694*
			(0.0378)	(0.0402)
Observations	12,555	12,555	12,555	12,555
R-squared	0.045	0.047	0.045	0.048
Control Variables	No	YES	No	YES
Province FE	YES	YES	YES	YES
Time FE	YES	YES	YES	YES

Standard errors in parentheses

*** p<0.01

** p<0.05

* p<0.1.

### 3.3 Are the measures necessary?

In more than half of the country the epidemic is not serious. For example, there is only one imported case in Tibet province, but all of the provinces adopted the level 1 response without exception. The implementation of prevention and control measures has major social and economic costs; thus, whether it is necessary to implement these strict large-scale measures throughout the country should be analyzed and discussed. According to the severity of the epidemic, we divided all cities in China into three groups: high, medium and low-risk areas. We defined areas with the number of confirmed cases below the 30th percentile of the number of confirmed cases on a particular day as low-risk areas, 30–60 percentiles as medium-risk areas, and those above the 60th percentile as high-risk areas, and then estimated the effectiveness of the measures separately. Table 6 reports the results of our sub-sample regression analyses. Our results show that the measures have the best effect in high-risk areas, followed by an effect in medium-risk areas, while both measures failed in low-risk areas. If these measures are implemented on a large nationwide scale without targeting, this would violate the principle of cost-effectiveness. Therefore, tailored measures should be implemented according to the specific situation of each area.

**Table 6:** The necessity of control measures in various areas.

	High-risk	Medium-risk	Low-risk	High-risk	Medium-risk	Low-risk
Traffic Control	-0.3325***	-0.1462***	-0.0323			
	(0.0394)	(0.0190)	(0.0243)			
Social Distancing				-0.2512***	-0.0948***	0.0044
				(0.0377)	(0.0188)	(0.0207)
Observations	7,298	3,076	2,181	7,298	3,076	2,181
R-squared	0.051	0.149	0.105	0.048	0.140	0.104
Control Variables	YES	YES	YES	YES	YES	YES
Province FE	YES	YES	YES	YES	YES	YES
Time FE	YES	YES	YES	YES	YES	YES

Standard errors in parentheses

*** p<0.01

** p<0.05

* p<0.1.

## 4. Conclusion and implication

In this paper, we applied econometric methods to construct an empirical model, and used the city-daily data from mainland China from January 1, 2020 to February 19,2020 to explore the role of prevention and control measures in the containment of the COVID-19 epidemic. We found that: 1. Both traffic control and social distancing have played a major role in controlling the development of the COVID-19 epidemic; 2. Traffic control is more effective than social distancing. The two measures are complementary, and their joint effect will play a stronger role in epidemic prevention; ③There is regional heterogeneity in the effectiveness of the prevention and control measures, and neither of the two measures is significant in areas with a low epidemic risk.

The results above have important implications for public health policy. First, we recognize the positive role of the measures taken by China in the fight against the COVID-19 epidemic, but the effects of these measures are highly heterogeneous, and neither of the two measures works in low-risk areas. But the implementation of these measures inevitably causes certain social and economic costs, for example, strict traffic control completely blocked population movement and logistics, which to some extent made it difficult for the timely replenishment of basic living needs. Social distancing measures, such as closing off communities and public places, inevitably exacerbated panic among people and adversely affected their mental health. Therefore, when implementing epidemic prevention and control measures, possible the negative impact should be considered, the need to implement such strict measures as a “lockdown” should be carefully weighted up, and the situation of each city should be fully taken into consideration to implement targeted and scientific epidemic control measures. Second, since the relationship between traffic control and social distancing measures are complementary, the two measures should be coordinated when policy-making to maximize their positive effects.

## Supporting information

S1 Data (DTA)
